# Diseases of Bones and Joints

**Published:** 1915-02

**Authors:** M. B. Grace


DISEASES OF BONES AND JOINTS. By Leonard W. Ely, M. D., Associate Profesosr of Surgery, Leland Stanford, Junior, University, San Francisco, Cal. Price, $3.00. Surgery Publishing Co., 92 William Street, New York, Publishers.

The general treatment of this subject by the ordinary textbooks, while taking up much time and space in detail, leave much to be desired by those who are really after the meat of the subject. To especially the general practitioner this little book will come as a boon, and should be on the table of every practicing physician and surgeon.

It is especially rich in photo-micrographs and plates serving to thoroughly illustrate the carefully written text, making it an exceedingly readable boo. While the subject is fully treated, the brevity and lucid manner of handling the subject matter gives the busy man just what he needs, yet stripped of the mass of details that usually go to make up burdensome reading.

The first chapter treats of the anatomy and histology of bone and joint tissues; their production, nutrition and repair as well as devoting some space to important general considerations.

The new illustrations assist in making the pathology especially plain and interesting and is followed by symtomatology, prognosis and the latest and more efficient methods of treatment.

When one has finished the book, he feels that he has been led through the laboratory and to the bedside by a competent teacher and has had the important points regarding these diseases impressed on his mind. M. B. Grace.

